# Choices in fluid type and volume during resuscitation: impact on patient outcomes

**DOI:** 10.1186/s13613-014-0038-4

**Published:** 2014-12-04

**Authors:** Alena Lira, Michael R Pinsky

**Affiliations:** 1 Department of Critical Care Medicine, University of Pittsburgh, 606 Scaife Hall, 3550 Terrace Street, Pittsburgh 15261, PA, USA

**Keywords:** Colloids, Crystalloids, Osmolality, Glycocalyx, Intravascular volume replacement, Systematic review

## Abstract

We summarize the emerging new literature regarding the pathophysiological principles underlying the beneficial and deleterious effects of fluid administration during resuscitation, as well as current recommendations and recent clinical evidence regarding specific colloids and crystalloids. This systematic review allows us to conclude that there is no clear benefit associated with the use of colloids compared to crystalloids and no evidence to support the unique benefit of albumin as a resuscitation fluid. Hydroxyethyl starch use has been associated with increased acute kidney injury (AKI) and use of renal replacement therapy. Other synthetic colloids (dextran and gelatins) though not well studied do not appear superior to crystalloids. Normal saline (NS) use is associated with hyperchloremic metabolic acidosis and increased risk of AKI. This risk is decreased when balanced salt solutions are used. Balanced crystalloid solutions have shown no harmful effects, and there is evidence for benefit over NS. Finally, fluid resuscitation should be applied in a goal-directed manner and targeted to physiologic needs of individual patients. The evidence supports use of fluids in volume-responsive patients whose end-organ perfusion parameters have not been met.

## Review

### Introduction

Fluid administration is perhaps the most ubiquitous therapeutic intervention in critically ill patients. There is a growing pool of evidence available to guide resuscitation and fluid administration practices. Some recommendations can be made unequivocally, while others continue to be subject of an ongoing discourse [1]-[3]. An interesting question arises from our review of the literature, as described below: Does practice follow the evidence? Specifically, in 2010, Finfer et al. [4] published results of a cross-sectional study conducted in 391 intensive care units across 25 countries around the world to evaluate fluid resuscitation practices. Their study revealed markedly desperate results. Despite available evidence, resuscitation practices varied significantly, with overall preference for the use of colloids. Importantly, choice of resuscitation fluid differed by country and took far less into consideration patients’ individual characteristics and available evidence than local practices. The practice is likely further shaped by economic considerations and local product availability [5]. Clearly, impressing evidence-based behaviors requires overcoming considerable traction of regional custom practices. Since the Finfer et al. study was conducted, multiple large high-quality prospective clinical trials have been published [Table 1] [6]-[10], and multiple meta-analyses [Table 2] [11]-[17] attempted to synthesize the evidence to help in developing clear consensus guidelines taking into consideration pathophysiological principles associated with resuscitation context as well as individual patient characteristics. This review will address these issues. As to the disparity between the evidence and practice, one only speculates why it exists. We reviewed the existing medical literature using both PubMed and Google Scholar search engines for the primary search terms such as clinical trial, fluids, resuscitation, crystalloids, and colloids and then expanded our search as linked citations indicated. We limited this search to studies published in English since the last Cochran Review on this subject [1].

**Table 1:** Characteristics of included randomized controlled trials

**Trial**	***N***	**Population**	**Type of fluid**				**Outcomes**	**Conclusion**
			**Intervention**	***N***	**Control**	***N***		
Myburgh 2012 (CHEST)	6,651	ICU patients	6% HES (130/0.4)	3,315	Saline	3,336	90-day mortality, AKI, RRT	No mortality difference; increased AKI and RRT use with HES
Perner 2012	798	ICU patients with severe sepsis	6% HES (130.0.42)	398	Ringer’s acetate	400	90-day mortality, RRT	Increased 90-day mortality with HES; increased use of RRT with HES
Yates 2013	202	Medium to high risk elective colorectal surgery patients	6% HES (130/0.4)	104	Hartmann’s solution	98	Day 5 post-op GI morbidity; post-op complications, LOS, coagulation and inflammation	No difference in any of the measured outcomes
Annane 2013 (CRISTAL)	2,857	ICU patients with hypovolemic shock	Colloids (gelatins, dextrans, HES, 4% or 20% albumin)	1,414	Crystalloids (isotonic or hypertonic saline, Ringer’s lactate)	1,443	28- and 90-day mortality; days alive without the need for RRT, MV, or vasopressors	No difference in 28-day mortality; 90-day mortality lower in colloid group
Caironi 2014 (ALBIOS)	1,810	ICU patients with severe sepsis or septic shock	20% albumin and crystalloid	903	Crystalloid solution	907	28- and 90-day mortality; organ dysfunction, LOS	No difference in mortality or other outcomes

*N*, number of patients; ICU, intensive care unit; HES, hydroxyethyl; AKI, acute kidney injury; RRT, renal replacement therapy; MV, mechanical ventilation; LOS, length of stay (ICU or hospital).

**Table 2:** Meta-analyses and systematic reviews

**Study**	**Number of trials**	**Number of patients**	**Population**	**Intervention**	**Control**	**Outcomes**	**Conclusion**
Serpa Neto 2014	10	4,624	Septic patients	HES	Crystalloids	28- and 90- day mortality, AKI, RRT, transfusion, LOS, fluid intake	HES shows increase in AKI, RRT, need for RBC transfusion, and 90-day mortality
Zarychanski 2013	38	10,880	Critically ill, including sepsis, trauma, burn, hypovolemic shock	HES	Crystalloids, gelatin, albumin	Mortality, AKI, LOS, MV	After exclusion of Boldt studies, HES increased mortality, AKI, and RRT
Gattas 2013	35	10,391	Critically ill or surgical patients	6% HES 130/0.4-0.42	Other fluids	Mortality, RRT, AKI, transfusion, bleeding	Increased risk of RRT with HES
Hasse 2013	9	3,456	ICU patients with sepsis	6% HES 130/0.38-0.45	Crystalloids or albumin	All cause mortality, RRT, AKI, bleeding and transfusion, adverse effects as defined in the individual studies	HES increased RRT, increased blood transfusion, increased incidence of adverse effects
Gillies 2013	19	1,567	Surgical patients	6% HES	Other colloids or crystalloids	Postoperative in hospital mortality, AKI, RRT	No difference in measured outcomes, no demonstrable benefit of HES
Perel 2013	70	22,392	Cochrane review 2013, critically ill	Colloids	Crystalloids	Mortality	Colloids do not decrease mortality, HES may increase mortality
Mutter 2013	42	11,399	Cochrane review	HES	Other fluids	Renal function	Increased need for RRT with all HES products in all patient populations
Bunn 2012	86	5,484	Critically ill and surgical patients in need of volume resuscitation, Cochrane review	Any one colloid (included albumin, HES, dextran, gelatin)	Any other colloid (included albumin, HES, dextran, gelatin)	Mortality, need for blood transfusion, adverse events	No benefit of one type of colloid over another
Thomas-Ruedel 2012	40	3,275	Adult and pediatric, primarily elective surgery, as well as ICU and ED	Gelatin	Albumin or crystalloid	Mortality, blood products administration, AKI, RRT	Unable to determine safety due to small studies and large heterogeneity
Rochwerg 2014	14	18,916	Adult patients with sepsis and septic shock	Any fluid (colloid or crystalloid)	Any fluid (colloid or crystalloid)	Mortality, blood products administration, AKI, RRT	Reduced mortality with balanced crystalloids and albumin compared to other fluids

ICU, intensive care unit; HES, hydroxyethyl starch; AKI, acute kidney injury; RRT, renal replacement therapy; MV, mechanical ventilation; LOS, length of stay (ICU or hospital); ED, emergency department.

Fluid administration is a vital component of resuscitation therapy in the hemodynamically unstable patient. Despite its ubiquity, however, for years this intervention remains a subject of an ongoing controversy. The discussion as to what fluid, how much, and when to give it was initially centered around the choice between colloid or crystalloid solutions, debating which was a better resuscitation fluid in terms of its ability to initially support intravascular volume and promote tissue perfusion, without causing interstitial edema [18]-[20]. Since all resuscitation fluids will expand the intravascular space to a greater or lesser degree [21], the debate now focuses more on the safety and efficacy of each particular fluid in resuscitation and improving longer-term patient outcomes. As newer colloid and crystalloid solutions entered the market, it became increasingly clear that differences in electrolyte composition and colloid particle size and composition had independent effects on these outcome measures [21]. The available colloids now include albumin, hydroxyethyl starch (HES), gelatin, and dextran. Available crystalloids include 0.9% normal saline (NS), lactated Ringer’s (LR) and its nearly identical brother Hartmann’s solution, and several similar balanced salt solutions (e.g., Plasma-Lyte, Normo-Sol). Not surprisingly, with more available clinical trial data, the debate behind fluid administration has expanded to include controversies surrounding particular solutions within each group, such as between HES versus albumin, and NS versus more balanced crystalloid solutions [22]-[24].

Importantly, for the following discussion, fluid administration needs to be placed in perspective. The practice of fluid administration in critically ill patients includes a variety of indications from simple replacement of insensible intravascular volume loss in patients unable to take fluids orally, replacement of volume deficits associated with hypovolemia or hemorrhage, to augmentation of volume in patients with pathology presenting with relative intravascular depletion, such as sepsis [25].

Historically, the administration of fluids directly into the circulation evolved to reverse severe dehydration resulting from volume loss due to diarrhea or vomiting in cholera patients [26]. The introduction of colloid came much later, during World War II, with infusion of albumin to maintain intravascular volume in trauma and severe burn patients [27]. The way in which fluids exert their therapeutic effects is by expansion of one of the three body volume compartments: intravascular, interstitial, and intracellular. The main goal for fluid resuscitation remains intravascular volume repletion from a functional hypovolemic state causing hemodynamic instability as manifested by end-organ hypoperfusion and extravascular volume depletion as manifested by dehydration and hyperosmolarity.

Initially, choices of a particular crystalloid solution were determined by availability and cost. For example, NS originally was less than half the cost of the other crystalloids and came in varying degrees of dilution (0.9, 0.45, and 0.225 N NaCl). It was also available alone or with 5% dextrose. Importantly, NS solutions are compatible with co-infusion of blood products. LR and other balanced salt solutions containing Ca^+2^ not only were more expensive, but also carried the risk of clotting infusion lines when blood transfusions were given. For all these reasons, the default crystalloid solution for resuscitation was NS except when hemostasis was needed, as often occurring in trauma or intraoperative resuscitation, wherein LR was usually prescribed [28]. These early concerns have now been minimized since all crystalloid solutions cost the same and multi-lumen infusion catheters simplify infusion compatibility concerns. Similarly, the choice of colloid solution remains influenced by cost and shelf life. The cost of albumin continues to vary considerably across countries and continents, and it has a limited shelf life, whereas starches and gelatins are cheaper and have a longer shelf life. Thus, economic considerations play an important role in determining which colloids are available regionally, but less so in the choice of crystalloids.

Without economic considerations, a decision on which is the optimal resuscitation fluid is often driven by the indications for fluid use and by what physiologic end-point needs to be targeted. Further consideration is often given to the underlying pathophysiology and how different fluid compositions will affect the *milieu interior*. Assuming that all other treatments are done similarly and correctly, one can then analyze the effectiveness and safety of specific fluid types in determining outcome. This presumption is difficult to accept, however, if the studies comparing one solution to another are retrospective chart reviews, or if prospective designs are unblinded or only partially blinded. Importantly, many new large multicenter clinical trials have provided insight as to the potential deleterious effects of specific types of solutions [29]-[33], making the choice of fluid less an academic exercise and more a therapeutic one.

### Basic pathophysiology and volume kinetics

A fundamental rationale for intravascular fluid resuscitation is to sustain an effective circulating intravascular volume or restore it to normal once initially depleted by hemorrhage or other causes of volume loss such as capillary leak, vomiting, diuresis, or diarrhea. These principles need to be taken into account when defining the clinical state as normovolemia, absolute or relative hypovolemia, and volume overload manifested as edema/anasarca.

Plasma water and solutes freely associate and move from the intravascular into the interstitial space at least once in a day owing to the greater hydrostatic pressure in the vascular space as compared in the interstitial space and the level of permeability of the vascular endothelium. This movement of fluid is increased to several times a day under pathophysiological conditions associated with systemic inflammation (trauma, sepsis) owing to increased capillary permeability [34],[35]. Fluid return to the vascular space is only minimal due to resorption back into the vascular system and mainly occurs by drainage back through the lymphatic system [36]. The balance of forces defining the rate of fluid transmission across the capillary endothelial barrier is described by the Starling equation:

$$J_{v}=K_{f}\left(\left[P_{c}-P_{i}]-\sigma [\pi_{i}-\pi_{c}\right]\right)$$

where *J*_v_ is the net fluid movement between compartments, [*P*_c_ − *P*_i_] − *σ* [*π*_i_ − *π*_c_] the net driving force, *P*_c_ the capillary hydrostatic pressure, *P*_i_ the interstitial hydrostatic pressure, *π*_c_ the capillary oncotic pressure, *π*_i_ the interstitial oncotic pressure, *K*_f_ the filtration coefficient for pressure-dependent fluid shifts, and *σ* the reflection coefficient for osmotically active vascular to interstitial gradient equilibration. Importantly, as will be discussed below, in many acute illnesses, both *K*_f_ and *σ* can decrease rapidly as the vascular endothelium’s glycocalyx is denuded [37],[38], making arguments as to which colloid or crystalloid solution will remain within the intravascular state mute.

Hydrostatic intravascular pressure is determined by both the upstream arteriolar resistance and downstream venous pressure, whereas hydrostatic interstitial pressure is determined primarily by tissue pressure. Gravitational pressure increases both venous pressure and tissue pressure equally, thus favoring hydrostatic translocation of fluid into the interstitium in dependent regions of the body. Oncotic pressure is determined by the solute concentration in the fluid. Since there is usually a higher concentration of solute in the plasma space owing to retention of albumin and other proteins such as globulins, normal oncotic pressure gradients promote reabsorption of interstitial fluid into the vascular space from the interstitial space [36]. The wild card in this balance is the relative resistance to fluid and solute flux across the semipermeable membranes of the vascular endothelium [39]. Under normal conditions, the endothelial membranes lining the capillaries are relatively impermeable with intercellular tight junctions holding neighbor endothelial cells together and the intravascular glycocalyx forming a protein barrier to solute flow [40]. These variables normally limit fluid flux in either direction [41]. However, adenosine triphosphate (ATP)-dependent transport mechanisms within the vascular lining endothelial cells promote significant solute transport across this barrier, such that in a steady state, there is a net loss of fluid into the interstitial space from the intravascular space which equals the entire circulating blood volume over a day [34].

Important in this process is the property that not all vascular beds have the same hydrostatic pressure or capillary permeability [42]. The splanchnic circulation has a greater degree of permeability than the muscle, brain, and kidney, owing to the hepatic sinusoidal structure. Thus, changes in blood flow distribution from splanchnic to muscle or vice versa will alter edema formation and the need for fluids to sustain normal homeostasis. Since anesthesia profoundly alters blood flow distribution, it also alters the steady-state balance of fluid resorption and lymphatic drainage [43],[44].

Disease states associated with inflammation like trauma, burns, sepsis, and acute pancreatitis are characterized by a marked reduction in the vascular endothelial glycocalyx [45]-[47]. The glycocalyx is the primary structure limiting free fluid flux across the vascular space [35]. Furthermore, if tissue injury also occurs, as often is the case in acute lung injury, vascular endothelial tight junction disruption will also occur in areas typically relatively resistant to fluid translocation, markedly opening up the interstitium to fluid translocation resulting in further imbalance between the intravascular and extravascular fluid and local interstitial edema [48]. Since different vascular regions of the body allow proteins to pass through the capillary membrane at different rates, as exemplified by the loose barrier in the liver and tight barrier in the brain, interstitial edema formation is not usually uniform throughout the body. Many of the plasma proteins in patients experiencing acute systemic inflammation will be cytokines and protein-bound hormones. Thus, the metabolic effect of differentially altered permeability and plasma leak may play a role in the regional expression of a generalized inflammatory response. Since most, if not all, fluid return from the extravascular space to the vasculature happens via lymphatic drainage [49], if transcapillary leakage is increased, the lymphatic system may become overwhelmed, further contributing to the development of edema and a relative intravascular volume deficit despite no actual loss of fluid outside the body. This fluid flux imbalance is accentuated further by slower lymphatic flow resulting from immobility in bedridden patients. Accumulation of intravascular fluid in the interstitial space is therefore dependent on multiple factors described in the Starling force equation above, permeability of the vascular membrane, as well as the capacity of the lymphatic system [50].

**Figure 1:**
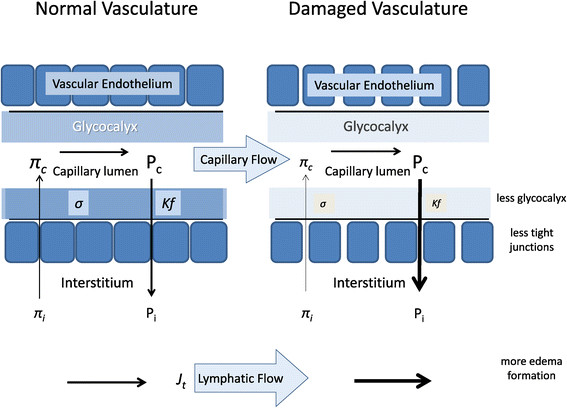
**Schematic diagram of the primary forces defining transcapillary fluid movement.** The opposing forces defining the steady-state net flow of fluid from the capillary into the interstitial space are defined by the hydrostatic pressure differences between the capillary lumen (*P*_c_) and interstitial pressure (*P*_i_) as opposed by the filtration coefficient (*K*_f_) which itself is a function of the vascular endothelial cell integrity and the intraluminal glycocalyx. This net efflux of fluid out of the capillary into the interstitium is blunted by an opposing oncotic pressure gradient moving fluid in the opposite direction because capillary oncotic pressure (*π*_c_) is greater than interstitial oncotic pressure (*π*_i_). And like hydrostatic pressure-dependent flow, oncotic dependent flow is blunted by the reflection coefficient (*σ*) which like *K*_f_ is a function of the glycocalyx and vascular endothelial integrity. Under normal conditions (left side), both *K*_f_ and *σ* are high minimizing fluid flux resulting in a slight loss of plasma into the interstitium which is removed by lymphatic flow. However, if the vascular endothelium and glycocalyx are damaged (right side), oncotic pressure gradients play a minimal role because a large amount of protein-rich plasma translocated into the interstitial space minimizing the oncotic pressure gradient, whereas the constant *P*_c_ promotes massive fluid loss and interstitial edema.

Based on the original Starling force concept, it made sense to use colloids with their higher oncotic pressure as a fluid resuscitation option, because in theory it would result in less capillary leak and edema formation while better supporting the intravascular volume needs. Regrettably, this theoretical model does not explain the observation that volume requirements during resuscitation in septic shock with either albumin or crystalloids are similar when both fluids are given in a blinded fashion [1],[51]. Indeed, even if the goal were to sustain a normal intravascular oncotic pressure, it has been repeatedly observed that the most balanced approach does not occur with infusion of colloids to crystalloids at a ratio of 1:3 as initially postulated [1],[52], but rather 1:1.3 [53]. This simplification is further complexed by the use of other synthetic colloids which when compared to albumin have different rates of degradation and half-life of elimination [34]. Furthermore, due to the increased capillary permeability in critical illness which results in accumulation of both fluid and macromolecules in the extracellular space, colloids may theoretically worsen edema by increasing interstitial oncotic pressure, resulting in further impediment of tissue perfusion and lymphatic return. This counterbalancing process as seen in otherwise healthy hypovolemia and inflammatory states is stylized in Figure 1.

Woodcock et al. [36] recently evaluated the basic physiological and molecular principles behind transvascular fluid exchange and called into question completeness of the initial Starling force principle. They propose a revised Starling model, which takes into account not only the composition of the intravascular fluid and the interstitial fluid, but also the physical characteristics of the transvascular barrier, which comprises of the endothelial glycocalyx layer and endothelial basement membrane, with tight junctions between cells and the extracellular matrix [40]. According to this revised model, when the vascular barrier is intact, transcapillary movement of fluid is unidirectional, as there is no absorption of fluid from the interstitium back to the intravascular space, and drainage of the interstitium is accomplished primarily by lymphatic clearance. Transcapillary movement is then dependent on capillary pressure. At supranormal capillary pressures, infusion of colloid solution preserves oncotic pressure and increases capillary pressure, thus increasing movement of fluid into the interstitial space. Under the same conditions, infusion of crystalloid solutions also increases capillary pressure, but by dilution decreases oncotic pressure, thus resulting in more transcapillary movement than colloids. At subnormal capillary pressures, transcapillary movement nears zero; thus, infusion of both crystalloids and colloids results in increase in capillary pressure, but no change in transcapillary movement.

The tissues that can accumulate large amounts of interstitial fluid during physiologic stress in their healthy state contain non-fenestrated capillaries. These include the liver and gut mucosa. These capillaries’ vascular endothelial barriers can undergo phenotypic changes from non-fenestrated to fenestrated, resulting in both endothelial dysfunction and increased permeability in response to physical and chemical stress. This change in the physical characteristics of the transcapillary barrier is largely responsible for increase in permeability leading to changes in volume kinetics, interstitial fluid accumulation manifested as edema, and its accompanying intravascular depletion.

This newly proposed mechanism explains why volume expansion with albumin in critically ill patients does not match that predicted by the Starling force model. Furthermore, it may explain why albumin has not been shown to provide benefit in volume expansion compared to crystalloids under conditions wherein endothelial glycocalyx disruption commonly occurs. The varying vascular permeability model underscores the integrity of the glycocalyx as one of the key factors involved in fluid dynamics and suggests that its restoration needs to be one of the therapeutic goals in conditions of physiologic stress [49],[50]. Regrettably, at this time, no specific therapies have been shown to augment glycocalyx restoration.

#### Crystalloids

We use the term ‘crystalloids’ to describe aqueous fluids that contain crystal-forming elements (electrolytes), which easily pass through vascular endothelial membrane barriers followed by water, leading to their equilibration between the intravascular and extracellular space. As noted above, in theory, this redistribution results in smaller retained intravascular volume of the initial infusion solution and development of edema when compared to colloid solutions [51]-[54].

Crystalloid solutions can contain a variety of inorganic cations, such as K^+^, Ca^++^, and Mg^++^, and organic anions, such as lactate, acetate, gluconate, or bicarbonate as well as Cl^−^, allowing the Na^+^, Cl^−^, and K^+^ values to vary independent of each other [Table 3]. The term ‘normal’ saline is a misnomer which was coined because its concentration is 0.9% *w*/*v* ‘normal’ or about 3,000 mOsm/L or 9 g/L, not because its composition is normal or ‘physiologic’ as an electrolyte solution. It is slightly hypertonic and has equal amounts of Na^+^ and Cl^−^, making it both hypernatremic and very hyperchloremic relative to the plasma. Thus, massive NS infusion will lead to hypernatremia and hyperchloremic metabolic acidosis and its associated sequelae such as renal vasoconstriction [55]. This reality by itself suggests that the best candidates for use of NS may be patients with the propensity for developing hyponatremia, hypochloremia, and metabolic alkalosis, as seen in severe persistent vomiting. Similarly, in patients in whom development of hyperchloremic metabolic acidosis could carry a significant morbidity (e.g., patients with compromised renal function or already existing acidosis), NS maybe contraindicated as a resuscitation fluid [56],[57]. Furthermore, blood ionic concentration changes can markedly alter pharmacodynamics. For example, studies in healthy volunteers demonstrated that when compared to solutions with lower chloride content, NS has slower excretion [55],[58].

**Table 3:** Characteristics of resuscitation fluids

**Solute**	**Plasma**	**Colloids**				**Crystalloids**			
		**4% albumin**	**6% HES 130/0.4**	**Dextran**	**Gelatin**	**Normal saline**	**Ringer’s lactate**	**Hartmann’s solution**	**Plasma-Lyte**
Na^+^	135 to 145	148	154	154	154	154	130	131	140
K^+^	4.0 to 5.0	0	0	0	0	0	4.5	5	5
Ca^2+^	2.2 to 2.6	0	0	0	0	0	2.7	4	0
Mg^2+^	1.0 to 2.0	0	0	0	0	0	0	0	1.5
Cl^−^	95 to 110	128	154	154	120	154	109	111	98
Acetate	0	0	0	0	0	0	0	0	27
Lactate	0.8 to 1.8	0	0	0	0	0	28	29	0
Gluconate	0	0	0	0	0	0	0	0	23
Bicarbonate	23 to 26	0	0	0	0	0	0	0	0
Osmolarity	291	250	286 to 308	308	274	308	280	279	294
Colloid	35 to 45	20	60	100	40	0	0	0	0

Osmolarity (mOsm/L); colloid (g/L); all other solutes (mmol/L).

Crystalloid alternatives to NS represent fluids more closely, resembling the electrolyte composition of plasma. The most frequently used ones are lactated Ringer’s solution or its nearly identical twin, Hartmann’s solution, and Plasma-Lyte (Table 3). Newer more balanced solutions are continuing to enter the market. LR has historically been used most frequently, but neither its ionic composition nor its tonicity is equivalent to that of plasma. Theoretically, discrepancies in tonicity can affect fluid distribution and pharmacodynamics-associated diuresis, both of which can have clinical implications [59]. Therefore, both the pharmacodynamics alterations due to tonicity and the metabolic effects of the ionic solute composition are of consideration when choosing between crystalloids.

#### Colloids

The term ‘colloids’ refers to aqueous solutions that contain both large organic macromolecules and electrolytes. Presumably, the large molecular size of the dissolved molecules limited their ability to cross the endothelial membrane. These molecules are retained within the intravascular space to a greater degree than pure crystalloids, owing to their higher oncotic pressure.

The first colloid solution used clinically was albumin. Albumin is harvested from human plasma. It is available in several concentrations (4%, 5%, 20%, and 25%). The greatest barrier to its use has been its cost, which varies widely across the world. Synthetic colloids, in particular starches (HES), gelatins, and dextran, present more economical alternatives. Gelatins are derived from bovine gelatin, and their colloid base is protein. HES are derived from the starch of potatoes or maize, and their colloid base is a large carbohydrate molecule. Solutions of various molecular weight are available, namely, 130, 200, and 450 kD. Dextran is also a carbohydrate-based colloid, a polysaccharide molecule made by bacteria during ethanol fermentation process. Oncotic pressure of these solutions varies depending on the molecular weight and concentration, and both hypo-oncotic (gelatins, 4% and 5% albumin) and hyper-oncotic solutions (20% or 25% albumin, dextran, and HES 6% and 10%) are available. The physiological actions, volume expansion properties, as well as potential morbidities of these solutions are determined by multiple factors which include oncotic pressure, molecular weight, half-life of degradation, chemical alteration of the macromolecules, and their tissue accumulations [60],[61]. The hydroxylation of starches, for instance, results in their accumulation in particular tissues including the skin, kidney, or liver, resulting in organ-specific clinical manifestations and potential morbidities such as acute kidney injury (AKI) or liver injury [62]-[64].

### Clinical evidence

#### Colloid versus crystalloid

##### Albumin controversy

The rationale behind using albumin and other colloids was driven by a theoretical assumption that colloids lead to better intravascular volume expansion compared to crystalloids. Though colloids result in transient greater increase in intravascular volume, it has not been shown that greater intravascular volume expansion translates to improvement in mortality outcomes. At the moment, no clear evidence exists to support widespread use of albumin resuscitation.

The early controversy on the use of albumin solutions in resuscitation was fueled by a Cochrane meta-analysis published in 1998, which showed that albumin use was associated with an increased mortality [65]. This meta-analysis used diverse data collated over decades and was of questionable validity. Importantly, the large multicenter SAFE trial, published in 2004 [66], showed no difference in mortality with the use of albumin versus NS, with the exception of the subgroup of traumatic brain injury (TBI) patients whose outcomes were worse with albumin [67]. FEAST study in 2011 [68] also showed no benefit of albumin over crystalloids. In that study, both colloids and crystalloids when used as a bolus in pediatric patients lead to increased mortality due to cardiovascular collapse. Not only did these studies show albumin did not increase mortality, but importantly, the SAFE study revealed in its subgroup analysis the use of albumin to be associated with decreased 28-day mortality in severe sepsis, suggesting a potential benefit of albumin use in this population in particular [69]. Recent meta-analysis by Rochwerg et al. resulting in composite global mortality risk comparisons of individual resuscitation fluids to one another in a multimodal analysis suggests that albumin is superior to other colloids, and its benefits over saline but not balanced crystalloids are supported by some studies with a moderate level of confidence [15].

The most recent clinical trial to address this issue was the ALBIOS trial [9] comparing 20% albumin to crystalloid in septic patient resuscitation. One thousand eight hundred patients with severe sepsis and septic shock were treated with either albumin and crystalloids, or crystalloids alone for 7 days (1:1 distribution). This trial showed that albumin-treated patients had significantly higher serum albumin level and had higher mean arterial pressure. However, these markers did not result in differences in mortality at 28 or 90 days. These data suggest that achieving higher perfusion pressures and oncotic goals does not equate improving survival. A *post hoc* subgroup analysis which looked at septic shock patients (>1,100 of the 1,800) showed that albumin-treated patients with septic shock did demonstrate decrease in mortality at 90 days, whereas the albumin-treated group in patients without septic shock had an increased mortality. Since this was a *post hoc* analysis, it is subject to bias and this data will require follow-up studies. Therefore, although the use of albumin does not portend harm, the evidence for its benefit does not exist, and at this time, the use of albumin in resuscitation of septic patients is not supported by clinical evidence.

Three additional clinical trials are currently underway attempting to answer the question of potential albumin benefit in sepsis, one of which is specifically looking at patients with septic shock. These trials are RASP (NCT01337934) evaluating use of LR compared to 4% albumin in patients with early sepsis, PRECISE (NCT00819416) looking at 5% albumin versus NS in early septic shock, and EARRS (NCT00327704) comparing NS to 20% albumin. Until these trials are completed, there is no evidence to show albumin to have any benefit over crystalloid solutions. Current guidelines and recommendations cannot endorse the use of albumin in light of its expense and current lack of proven benefit.

##### Hydroxyethyl starch controversy

Synthetic colloids are often used in resuscitation, especially in the operating room and outside of North America. Multiple studies and recent meta-analyses evaluated the outcomes associated with the use of synthetic colloids, showing no benefit of individual synthetic colloids over other colloids or over crystalloids [7],[8],[10],[14],[15]. Larger debate has emerged surrounding HES in particular, with controversy compounded, by the discovery that many of the original data published by Joachim Boldt showing outcome benefits of HES were falsified, resulting in subsequent retraction of these studies [70],[71]. The lack of mortality benefit of HES has been shown in several large recent randomized control trials (RCTs). Perner et al. in 2012 [7] showed increase in 90-day mortality with HES when compared to LR in 800 patients with severe sepsis. The CHEST trial [8] showed no difference in mortality between HES and NS in a 7,000 patient general intensive care unit (ICU) population, and Bagshaw et al. [10] showed no mortality difference in a 7,000 patient multicenter RCT comparing HES to NS. Similarly, a study evaluating goal-directed fluid therapy in colorectal surgery showed no mortality benefit of HES over balanced crystalloid solution [69]. Three recent meta-analyses by Zarychanski et al. [12], Serpa Neto et al. [13], and Rochwerg et al. [15] support the conclusion that use of HES in resuscitation does not reduce mortality when compared to other resuscitation fluids. To the contrary, some studies suggest increase in mortality with HES [7], and after exclusion of studies by Boldt, the meta-analysis by Zarychanski et al. also showed a similar non-significant trend toward HES causing harm.

Contrasting this is the recent CRISTAL trial, which enrolled 2,857 patients with hypovolemic shock, sepsis, and trauma in multiple centers from five different countries [6]. This trial compared administration of colloids (hypo- as well as hyper-oncotic) to crystalloids (including isotonic and hypertonic saline as well as balanced solutions) and detected a difference in 90-day mortality, favoring the use of colloids. HES was the most commonly used colloid (used in 70% of patients in the colloid group), and NS was the most commonly used crystalloid (used in 80% of patients in the crystalloid group). The study observed no difference in 28-day mortality between treatment groups. They also found no difference in the need for renal replacement therapy between groups. It is unclear why the results of this large study differ from other large recent studies but may be attributed to several peculiarities in the actual treatment each group received. The data was analyzed based on intention to treat analysis. However, many deviations from the assigned treatment were noted in both the colloid and crystalloid groups. Further confounding may have resulted from the period prior to randomization, when many patients received resuscitation fluids different from those to which they were then assigned upon randomization. Furthermore, given that the data regarding HES varies with variables such as molecular weight in ways that is not consistent with any particular hypothesis, this suggests that other confounding factors may exist that are not being accounted for. One of such confounding factors may in fact be the electrolyte composition of the solution used for preparation of the starches.

Although the HES controversy still surrounds mortality, there is significant evidence that HES increases morbidity. Its use has been shown to result in increase in serum creatinine and increased use of renal replacement therapy (RRT) both in clinical trials [7],[8] and meta-analyses [10]-[17]. Although the results of these meta-analyses were driven primarily by the large trials, they also included numerous smaller studies, confirming this finding of HES association with increase in AKI and need for RRT. One meta-analysis [22] did not show such association, but it is difficult to interpret its findings because it compared HES to any other resuscitation fluid in surgical patients, including 19 small studies of which only three compared HES to crystalloids and reported on AKI. Interestingly, none of the meta-analyses included the CRISTAL trial, and again, the CRISTAL trial data did not support the findings of increased need for RRT with HES, in contrast to the other large trials such as CHEST [8] and 6S [7]. There are a few differences that distinguish these trials. These include using as the comparison group LR or NS, the clinical acuity of the enrolled patients, the pre-randomization fluid type and volume, as well as use of maintenance fluids. We now know that in a particularly susceptible population, NS may have some deleterious effects on the kidney, which until recently were underappreciated. It is possible that the HES versus LR comparison and HES versus NS comparison do not yield the same results. The level of clinical acuity may deem a patient susceptible to developing clinically relevant AKI in the setting of an offending agent, and furthermore, once AKI develops, it may be more poorly tolerated, posing a greater contributor to mortality. There may be additional patient susceptibility factors that also provide a biologically plausible explanation for the differences between the studies’ outcomes which are not presently obvious.

Debate raised by these studies addresses questions of superiority of crystalloids versus colloids, and benefits and drawbacks of the use of HES in comparison to other fluids. The discussion largely explores the question of equivalence of colloid solutions, but it does not address the question of equivalence of the different crystalloid solutions. Crystalloids were assumed to be equal and by bias equally bad at restoring intravascular volume, when the study designs were initially created. Those large trials which did not support differences in mortality or renal injury between synthetic colloids and crystalloids, namely, those by Bagshaw et al. and Annane et al. [6],[10], compared HES to NS. This raises the question whether the choice of crystalloid solution may play a role in the mortality and morbidity outcomes. If NS carried its own mortality and morbidity effect, then the studies using NS in the crystalloid arm might not reflect actual colloid versus crystalloid difference in mortality and morbidity. In light of the complexity that continues to emerge, the original question of choosing between colloids versus crystalloids may need to be rephrased. Owing to the fact that albumin appears superior to other colloids and balanced solutions are different from NS, comparing colloids to crystalloids as groups becomes less informative than initially thought.

#### Chloride-liberal versus chloride-restricted crystalloids

The above question brings into focus the need for head-to-head comparison of the different crystalloid solutions. There has been a recent surge in the literature comparing different crystalloid solutions in resuscitation, in particular chloride-liberal (i.e., NS) versus chloride-restricted solutions [32],[72]. These results come primarily from perioperative literature including mainly trauma patients and inpatients undergoing major abdominal surgery and suggest that the use of balanced salt solutions in some patient populations decreases mortality and the incidence of AKI when compared to NS. This provoked a more rigorous evaluation of the effects of NS in comparison to balanced crystalloids in critically ill patients [33],[73]. A clinical trial by Yunos et al. involving 2,012 patients demonstrated a decreased AKI incidence and use of RRT in ICU patients with implementation of chloride-restricted strategy [33]. The use of NS has long been known to be associated with an increased risk of hyperchloremic metabolic acidosis [74], but it has only recently been shown that these metabolic changes can result in decreased renal blood flow and renal cortical hypoperfusion, as demonstrated in healthy volunteers [55]. Several studies now have shown perioperative mortality and morbidity benefits of balanced solutions over NS, and growing evidence exists suggesting greater benefit in critically ill patients [32],[33],[75],[76].

#### Goal-directed therapy

Although not the focus of our review, aside from the type of fluid, consideration should be given to the amount of fluid during resuscitation and its timing relative to the exogenous stress. Much of the approach to the decisions regarding volume of fluid resuscitation comes from perioperative literature [77]-[81]. Recent studies on perioperative fluid administration challenge prior usual practices and suggest that significant benefit can be achieved by individualizing therapy based on patient response. Perioperative fluid management has long been dictated by the generalized formulaic approach, rather than physiologic and homeostatic needs [82]. However, both perioperative fluid under-resuscitation as well as over-resuscitation can have deleterious effects and lead to increased morbidity and mortality [78],[81],[83].

Goal-directed fluid resuscitation therapy targets physiologic goals of hemodynamic stabilization, and benefit of such approach has been shown in multiple studies and recent meta-analyses [84]-[86]. The main goal of such therapy is maintenance of end-organ perfusion, achieved by adequate circulating volume as well as adequate function of the cardiovascular system. All of these components can be altered perioperatively by anesthetic agents, body temperature, or other factors. Thus, fluid resuscitation should be used to achieve these specific goals when monitoring suggests the patient to be fluid responsive [87],[88]. The counterargument is raised by studies evaluating fluid resuscitation in the septic patient, where the field has been driven by the Surviving Sepsis Campaign [89], and further addressed by the recent ProCESS and ARISE trials [86],[90]. Interestingly, the recently completed ProCESS trial showed no difference in outcome of sepsis patients treated in an emergency department with early goal-directed therapy versus two types of usual care. Importantly, all three arms of that study received roughly the same amount of fluid therapy both in the initial few hours and over the first day [90]. Recent analysis by Wachter et al. has looked at the interplay between volume resuscitation and use of vasopressors [91] and maintains that volume resuscitation is critical in septic patients in the early phase of the illness. Also, the patients who exhibited lowest in hospital mortality were the ones who received moderate to high fluid volume in the first 6 h of resuscitation, but delayed vasopressor use until adequate volume resuscitation has been obtained.

The other recent trial in the septic population is the ARISE trial [86]. Here investigators compared the implementation of early goal-directed therapy with ‘standard’ practice and found that patients in the early goal-directed therapy (EGDT) arm received higher resuscitation fluid volume and higher amount of vasopressors, and were noted to have higher blood pressures, but this finding did not translate into change in 90-day mortality, suggesting that goal-directed therapy in early sepsis does not yield survival benefit.

Although seemingly in contrast to one another and to the perioperative literature, we believe that the overall message is the same once interpretation is taken in context. First, it must be questioned whether a decade of EGDT has changed our practice to the point that our ‘standard’ practice has moved away from formulaic approaches, and favors more aggressive volume and vasopressor resuscitation based on physiological principles. Second, we note that in the perioperative literature, the goal-directed approach to resuscitation typically results in more conservative volume resuscitation, whereas in septic patients, the goal-directed approach results in greater volume administration, suggesting that the goal-directed approach potentially unmasks greater volume needs in patients in early sepsis as their physiology progresses into a more distributive and vasodilatory state. In contrast, intraoperative resuscitation may be reflective of a state that is more vasoconstrictive owing to a combination of different anesthetic agents, pharmacologic vasogenic agents, and intraoperative hypothermia.

Hence, it is probably not just the amount of volume, but mostly the ability to stabilize the critically ill patient with that volume that defines outcome [92],[93]. Volume responsiveness is only one of the components of the perioperative or septic physiologic state, the others being need and responsiveness to vasoactive agents and inotropic support. Therefore, fluid resuscitation therapy should not be used in isolation since the goals of therapy are to make the patient cardiovascularly sufficient. Clearly, expert knowledge of understanding the pathophysiologic principles and how they contribute to each individual’s acute pathophysiologic state, the type of surgical procedure or stress, and unique underlying comorbidities needs to be incorporated into the treatment plan [79]. Fluid therapy therefore should be used only in volume-responsive patients and only when end-organ perfusion goals are not met. Of note is that it is not sufficient to target volume administration to arterial blood pressure, as recent study by Asfar et al. showed that improved arterial blood pressure was not necessarily associated with better outcomes [94]. Hence, determining fluid need is dependent on dynamic parameters of hemodynamic monitoring and should be individualized to each patient [95]. Studies comparing goal-directed fluid administration strategies with fluid-liberal strategies show improved outcomes with goal-directed therapies [96]-[98].

## Conclusions

Keeping with the current evidence, organizations and collaborations such as the European Society of Intensive Care Medicine (ESICM) and Cochrane umbrella the spectrum of findings under consensus statement and create a set of recommendations [1],[3]. In summarizing these most recent recommendations and with addition of the literature that emerged over the past couple of years, the following conclusions can be drawn.

1. *Colloids at large*: There has not been a clear benefit associated with the use of expensive colloids compared to inexpensive crystalloids. Colloids as a whole have, however, shown to have increased mortality in patients with TBI. No indications currently exist for the routine use of colloids over crystalloids.

2. *Albumin*: There is no evidence to support the unique benefit of use of albumin as a resuscitation fluid. With the inclusion of the latest ALBIOS trial, mortality benefit in sepsis has not thus far been proven. In light of the cost and limited shelf life, the use of albumin as a resuscitation fluid is not supported.

3. *HES*: The benefit of using HES has been refuted. To the contrary, HES is associated with increased harm. Though it is not clearly associated with increase in mortality, evidence clearly shows increased AKI and use of RRT associated with the use of HES. It is further associated with coagulopathy and increased use of blood transfusion. The effects seem to be dose dependent, but no consensus has been reached as to a safe dose of HES. As such, the use of HES in resuscitation should be avoided.

4. *Dextran and gelatins*: Other synthetic colloids (dextran and gelatins) are not well studied in the literature. Although there is no evidence showing harm beyond what is seen with other colloids, there is also no evidence showing benefit. In light of the lack of evidence, and the theoretical potential for adverse effect, the suggestion is not to use gelatins or dextran.

5. *0.9% saline*: The use of NS has been shown to be associated with development of hyperchloremic metabolic acidosis and increased risk of AKI in susceptible patients, especially those with diabetic ketoacidosis. This risk is decreased when balanced salt solutions are used. The use of balanced crystalloid solutions rather then NS when possible should be considered in these populations.

6. *Balanced crystalloid solutions*: These solutions have shown no harmful effects in any particular patient population. There is evidence for benefit over NS as a means of preventing development of hyperchloremic metabolic acidosis and its associated effects. There is no head-to-head study comparing different balanced crystalloids to each other, and therefore, no consensus exists on a single preferred solution. Current literature supports use of balanced crystalloids when possible and in particular in patients in which NS may cause adverse effects, as mentioned above.

7. *Volume*: Fluid resuscitation should be applied in a goal-directed manner and targeted to physiologic needs of individual patients. The evidence supports use of fluids in volume-responsive patients whose end-organ perfusion parameters have not been met. Studies show improved outcomes with the use of goal-directed therapy over fluid-liberal approach.

## Abbreviations

AKI: acute kidney injury

ATP: adenosine triphosphate

ESICM: European Society of Intensive Care Medicine

HES: hydroxyethyl starch

ICU: intensive care unit

*J*_v_: net fluid movement between compartments

*K*_f_: filtration constant

LR: lactated Ringer’s

NS: normal saline

*P*_c_: capillary hydrostatic pressure

*P*_i_: interstitial hydrostatic pressure

RRT: renal replacement therapy

TBI: traumatic brain injury

*π*_c_: capillary oncotic pressure

*π*_i_: interstitial oncotic pressure

*σ* : reflection coefficient

## Competing interests

The authors declare that they have no competing interests.

## Authors’ contributions

AL performed the systematic review searches and reviewed the primary manuscripts cited in this review, wrote the initial draft of the manuscript, and contributed to revisions of the final version. MP reviewed the initial search results and all the primary manuscripts cited in this review, and revised and wrote the final version of the manuscript. Both authors read and approved the final manuscript.
